# Associations between environmental factors and incidence of cutaneous melanoma. Review

**DOI:** 10.1186/1476-069X-11-S1-S12

**Published:** 2012-06-28

**Authors:** Katarina Volkovova, Dagmar Bilanicova, Alena Bartonova, Silvia Letašiová, Maria Dusinska

**Affiliations:** 1 Slovak Medical University, Bratislava, Slovakia; 2 University of Venice, Venice, Italy; 3 NILU - Norwegian Institute for Air Research, Oslo, Norway; 4 Slovak University of Technology, Bratislava, Slovak Republic

## Abstract

**Background:**

Cutaneous melanoma is one of the most serious skin cancers. It is caused by neural crest-derived melanocytes - pigmented cells normally present in the epidermis and, sometimes, in the dermis.

**Methods:**

We performed a review of current knowledge on the risk factors of cutaneous melanoma. Relevant studies were identified using the PubMed, Science Direct, Medline, Scopus, Scholar Google and ISI Web of Knowledge databases.

**Results:**

Melanoma incurs a considerable public health burden owing to the worldwide dramatic rise in incidence since the mid-1960s. Ultraviolet radiation exposure is the predominant environmental risk factor. The role of geographical (latitude) and individual factors such as skin type, life style, vitamin D levels and antioxidant protection, sunburn, and exposure to other environmental factors possibly contributing to melanoma risk (such as cosmetics including sunscreen, photosensitising drugs, and exogenous hormones) are reviewed in this article. Recently, both rare high risk susceptibility genes and common polymorphic genes contributing to melanoma risk have been identified.

**Conclusions:**

Cutaneous melanoma is a complex cancer with heterogeneous aetiology that continues to increase in incidence. Introduction of new biomarkers may help to elucidate the mechanism of pathogenesis and individual susceptibility to the disease, and make both prevention and treatment more effective.

## Background

Cutaneous melanoma is one of the most serious skin cancers. It is caused by neural crest-derived melanocytes - pigmented cells normally presented norma in the epidermis and, sometimes, in the dermis [1]. Incidence of melanoma is increasing. While in US the lifetime risk of melanoma in 1935 was one in 1 500 persons, in 1960 it was one in 600 persons, and in 2000 it was one in 75 persons [2]. The progression of the melanocyte to a malignant melanoma involves various sequential steps: development of benign naevocellular naevus preneoplastic dysplastic naevus primary melanoma and metastatic melanoma [3]. There are four main types of cutaneous malignant melanoma [4,5]: a) Superficial spreading malignant melanoma which is the most common among Caucasians and accounts for 70 percent of all melanomas. It usually occurs in adults and may develop anywhere on the body but appears with increased frequency on the upper back of both men and women and on the legs of women; b) Nodular melanoma (accounting for 15 to 30 percent of all melanomas), a dome-shaped, pedunculated or nodular lesion that may occur anywhere on the body. It is commonly dark brown or reddish brown but may occasionally be amelanotic. Nodular melanomas tend to rapidly invade the dermis from the onset with no apparent horizontal growth phase. These tumors are frequently misdiagnosed, because they may resemble blood blisters, hemangiomas, dermal nevi or polypi; c) Lentigo maligna melanoma (which accounts for 4 to 10 percent of all melanomas) originates from lentigo maligna. Untreated lentigo maligna tends to exhibit horizontal or radial growth with epidermal involvement for many years (often decades) before it enters the vertical growth phase and invades the dermis to become lentigo maligna melanoma. This change is often indicated clinically by the development of focal papular or nodular areas; d) Acral lentiginous melanoma (2 to 8 percent of all melanomas) occurs on the palmar and plantar surfaces, the digits and the subungual areas.

Although the prognosis of thin melanoma is relatively good, prognosis decreases with increased thickness of the lesions. The diminished prognosis is mainly due to the well established tendency of melanoma to metastasize, which accounts for 75 percent of all deaths associated with skin cancer. In addition, melanomas are highly resistant to most forms of chemotherapy and radiation; therefore, cure of the disseminated disease is uncommon [6]. In women, melanoma often develops in the extremities, most commonly the lower limbs. In men, melanoma is most often found on the trunk, on the area between the shoulders and hips. In both sexes, melanoma can appear on the palms or soles and under the fingernails or toenails [7].

## Methods

An extensive literature search was performed to review current knowledge on cutaneous melanoma including epidemiological studies addressing risk factors of cutaneous melanoma between 1998 and 2010. Relevant studies were identified using the PubMed, Science Direct, Medline, Scopus, Scholar Google and ISI Web of Knowledge databases.

## Results – Melanoma risk factors

### Sunlight exposure

Sun exposure plays a primary and supporting role in most melanoma tumors. There is evidence that for the four main cutaneous types of melanomas, the pattern of excess sunlight exposure which is most damaging varies [8,9,5]. The environmental human carcinogen present in sunlight is ultraviolet (UV) irradiation [10]. The sun emits UVA (λ=320-400 nm), UVB (λ=280-320 nm), and UVC (λ=200-280 nm) ultraviolet radiation. While UVC radiation is ecologically not relevant since it is absorbed by oxygen and ozone in the Earth’s atmosphere, the longer wavelength UV-B (280–315 nm) and UV-A (315–400 nm) radiation have significant effects on the biota. 98.7% of the ultraviolet radiation that reaches the Earth's surface is UVA [11,12]. The molecular mechanisms by which UV radiation exerts its varied effects are not fully understood; however, it is thought that UV irradiation plays a critical role in melanoma formation [1]. Currently, it is thought that the DNA damaging, carcinogenic, inflammatory, and immunosuppressive properties of UVR all contribute to initiation, progression, and metastasis of primary melanoma [13]. Reactive oxygen species (ROS) overproduction may stimulate malignant transformation to melanoma. Changes in ROS signalling pathways play also important role in the damaging action of UVA and UVB irradiation on the skin [14].

### Natural photoprotection

The term photoprotection designates the mechanisms that nature has developed to minimize the damage that the human body suffers when exposed to UV-irradiation. This damage mostly occurs on the skin, but other parts of the body (especially the testicles) can be affected by UV-light. Photoprotection of the human skin is achieved by the extremely efficient internal conversion of molecules which originally absorb the UV-photon - endogenous chromophores: DNA nucleotides, urocanic acid, proteins, amino acids, melanin and their precursors and metabolites [15]. Internal conversion is a photochemical process that converts the energy of the UV-photon into small amounts of heat. These small amounts of heat are harmless. The energy of the UV-photon not transformed into heat leads to the generation of various harmful reactive chemical species (e.g. singlet oxygen or hydroxyl radical) [16,17].

**Figure 1:**
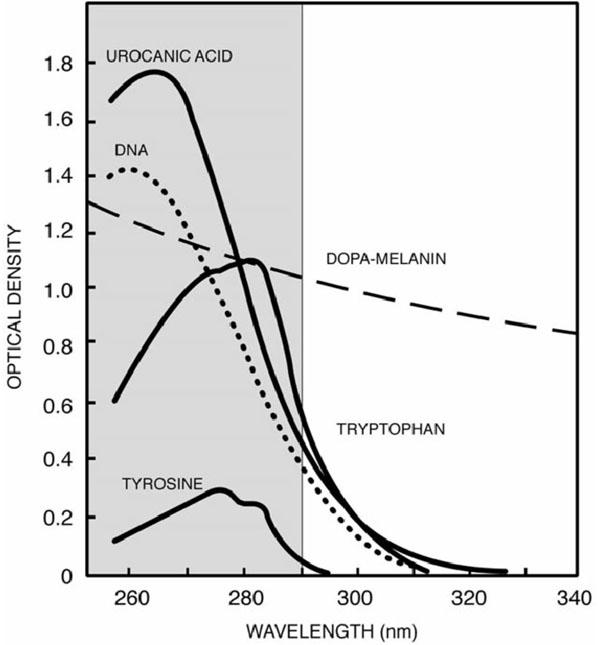
**UVR absorption spectra of molecules important to UV-induced health effects** DOPA-melanin-synthetic model of eumelanin (J Longstreth et al, 1998)

In DNA this photoprotective mechanism evolved four billion years ago. The purpose of this extremely efficient photoprotective mechanism is to prevent direct and partially indirect DNA damage. The ultrafast internal conversion of DNA reduces the lifetime of DNA in the excited state to only a few femtoseconds (10^-15^s) – in this way the excited DNA does not have enough time to react with other molecules [18,19,17]. The absorption spectrum of DNA shows strong absorption for UVB-radiation and much lower absorption for UVA-radiation (Fig. 1).

It is thought that photoprotection of melanin developed later in the course of evolution. There are two types of skin melanin: eumelanin, a black-brown pigment which is insoluble and is found in brown/black hair and brown eyes, pheomelanin, reddish pigment which is alkali-soluble and is found in red hair and red feathers [20]. All healthy individuals have varying degrees of eumelanin in their skin, while pheomelanin is present only in individuals who carry the corresponding genetic trait. It is thought that eumelanin protection against nutrient photolysis and, specifically, photolysis of folate (owing to the direct connection between folate and reproductive success), was a prime selective inducer which resulted in deeply pigmented skins among people living under high UVB radiation throughout most of the year. The importance of increased eumelanin production to prevent future direct and indirect DNA damage, individual fitness of protection of sweat glands and maintenance of thermoregulatory capability is also thought to have contributed to increased melanization [21]. Eumelanin dissipates more than 99.9% of the absorbed UV radiation as heat. This means that less than 0.1% of the excited melanin molecules will undergo harmful chemical reactions or produce free radicals [22].

### DNA damage

The predominant component of sunlight, UVA, penetrates fivefold deeper into skin than UVB due to its longer wavelengths [23,24]. Whereas UVA can indirectly damage DNA through the formation of reactive oxygen radicals, UVB can directly damage DNA causing apoptosis of keratinocytes by forming sunburn cells [25]. It has been shown that 92% of all melanoma are caused by indirect DNA damage and that only 8% of all melanoma are caused by direct DNA damage [26].

### Direct DNA damage and sunburn

Since the action spectrum of sunburn is identical to the absorption spectrum of DNA, it is generally accepted that direct DNA damage is the cause of sunburn [27]. It can occur when DNA directly absorbs UVB photons. UVB light causes the formation of covalent linkages between pairs of thymine and cytosine bases in DNA and forms pyrimidine dimers (cyclobutane dimer). The radiation excites DNA molecules in skin cells, causing aberrant covalent bonds between adjacent cytosine bases by producing a dimer. During DNA replication, DNA polymerase incorporates an incorrect base opposite to an aberrant base, causing a mutation. The mutation caused by direct DNA damage can lead to skin cancers. The second most frequent UV photoproducts are 6-4 photoproducts (6-4 PPs), which are pyrimidine adducts and their Dewar valence isomers formed by the photo isomerisation of 6-4 PPs by wavelengths longer than 290nm [28-33]. These reactions are quite common: each cell in the skin might experience 50-100 reactions during every second of sunlight exposure. Most of these genetic lesions are corrected by the mechanism of nucleotide excision repair. If the damage remains uncorrected, the genetic information may be permanently mutated.

### Indirect DNA damage and melanoma

Malignant melanoma is mainly caused by indirect DNA damage. A limited number of molecules in tissue weakly absorb UVA irradiation. After UVA irradiation absorption, these molecules (endogenous photosensitizers) converts to their long-lived triplet state that allows the transfer of energy to oxygen molecules. The transferred energy leads to an energetically excited oxygen molecule (singlet oxygen), which is highly reactive. Some of the endogenous photosensitizers have been identified, for example, flavins [34], NADH/NADPH [35], urocanic acid [36], and some sterols [37]. 3-hydroxypyridine derivatives comprising a wide range of skin biomolecules, such as enzymatic collagen cross-links, B6 vitamin, and likely advanced glycation end products in chronologically aged skin, constitute a novel class of UVA photosensitizers, capable of skin photooxidative damage [38]. Wenczl et al. [39] demonstrated that UVA-irradiated cultured human melanocytes are photosensitized also by chromophores as pheomelanin and/or melanin intermediates. UVB natural chromophores may also exhibit similar phototoxic properties. Babu and Joshi [40] suggested that UVB-sensitized tryptophan produces singlet oxygen (^1^O_2_) and superoxide radicals (O_2_^-^.), and these reactive forms of oxygen may contribute to membrane-, cytoplasm- and DNA-damaging effects.

Singlet oxygen, hydroxyl radical and hydrogen peroxide are reactive oxygen species (ROS) considered to be most responsible for producing oxidative stress in cells and organisms [41]. Oxidative stress is caused by imbalance between ROS production and a biological system's ability to readily detoxify these reactive intermediates or easily repair the resulting damage. Thus, oxidative stress is accepted as a critical pathophysiological mechanism in cancererogenesis [42]. Reactive chemical species can reach DNA by diffusion and the bimolecular reaction will damage the DNA [43]. Singlet oxygen interacts preferentially with guanine to produce 8-oxo-7,8-dihydroguanine (formed by the loss of two electrons). Further removal of two electrons from this product can yield spiroiminodihydantoin (Sp) R and S stereoisomers. Both in vitro and in vivo experiments have shown that the Sp stereoisomers are highly mutagenic, causing G --> T and G --> C conversions. Hence, they are of interest as examples of endogenous DNA damage that may initiate cancer [41,44].

### Ozone depletion and skin cancer

The radical increase of melanoma that has occurred in the last years has been associated with ozone depletion caused by ozone depleting substances (ODS) of anthropogenic origin, resulting in higher UVB radiation reaching the ground. Decreases in ozone have generated worldwide concern leading to adoption of the Montreal Protocol banning the production of ODS such as chlorofluorocarbon (CFC), other fully halogenated CFCs (freons), carbon tetrachloride, hydrochlorofluorocarbons, hydrobromofluorocarbons, methylchloroform, important industrial substances like halons (bromofluorocarbon compounds) and methyl bromide. Ozone depleting substances were used in automobile and truck air conditioning units, domestic and commercial refrigeration and air conditioning/heat pump equipment, aerosol products, portable fire extinguishers, insulation boards, panel and pipe covers and pre-polymers (The Montreal Protocol on Substances that Deplete the Ozone Layer, 2000). The level of UVB in sunlight is a strong function of latitude, whereas UVA is not. It is not surprising that the ratio of non-melanoma skin cancer incidence in Australia/Norway is an order of magnitude higher than for cutaneous malignant melanoma [45]. Based on studies which have found that 92% of all melanoma are caused by indirect DNA damage from UVA [46], we assume that other main hazard factors (pigmentary traits, ethnic origin, benign nevi, or family history) and not ozone depletion are responsible for radical increase of melanoma last years.

### Sunburn and sunscreens

An increased risk of melanoma was seen with increasing number of sunburns for all ages, not just childhood. The magnitude of risk for five sunburns per decade was shown to be highest for adult and lifetime sunburns [47].

Although sunscreens prevent sunburn [48-50], epidemiological or laboratory evidence that they prevent melanoma in humans is still missing.

Sunscreens have traditionally been divided into organic (chemical) absorbers and inorganic (physical) blockers on the basis of their mechanism of action. The organic compounds absorb high- intensity UV rays with their excitation to a higher energy state. Excess energy is dissipated by emission at longer wavelengths or relaxation by photochemical process such as isomerization and heat release. These organic compounds include para-aminobenzoic acid (PABA) and PABA esters, salicylates, cinnamates, benzophenons, butyl methoxydibenzoylmethane (Parsol 1789), drometrizole trisulphonic (Mexoryl XL), terephthalydene dicamphor sulphonic acid (Mexoryl SX), methylene bisbenzotriazol tetramethylbutylphenol (tinasorb M) and anisotriazine (Tinasorb S).

The inorganic agents, which protect the skin by reflecting and scattering UV, are nanoforms of titanium dioxide (TiO_2_) and zinc oxide (ZnO). These sunscreens are very efficient, photostable and offer protection extending into the UVA and visible range of the electromagnetic spectrum with almost negligible irritation. However, these molecules which reflect/scatter UV can cause whitening of the skin. Therefore, metal oxides are now frequently processed as microfine or nanoparticles (10-50 nm). Nanoparticles reflect/scatter and absorb UVA and UVB, and they are transparent on the skin, thus enhancing the cosmetic acceptability of the product [51].

It was thought that the UV-filter acts as "artificial melanin" but most of sunscreen organic chemicals cannot dissipate the energy of the excited state as efficiently as melanin or DNA (Table.1) and, therefore, the penetration of sunscreen ingredients into the lower layers of skin increases the amount of ROS [52,53].

**Table 1:** Dissipation of photon energy by natural and synthetic organic chromophores

UV-absorber	Other names	Percentage of molecules that dissipate the photon energy
**DNA**		> 99.9 %

**natural melanin**		> 99.9 %

**2-ethylhexyl 4-(dimethylamino) benzoate**	Padimate-O, octyldimethyl PABA, OD-PABA	10%

**4-Methylbenzylidene camphor**	(4-MBC), (MBC), Parsol 5000, Eusolex 6300	30%

**Menthyl anthranilate**	(MA), Methyl-2-aminobenzoate, meradimate	60%

**Ethylhexyl methoxycinnamate**	(2-EHMC), (EHMC), EMC, Octyl methoxycinnamate, OMC, Eusolex 2292, Parsol	81%

Inorganic sunscreen agents (metal oxides) screen UVA/UVB radiation efficiently, but can also generate harmful reactive oxygen species and radicals when subjected to UVA/UVB radiation [54,55].

DNA damage can be reduced by topical sunscreen which stays on the surface of the skin; it is important that the sunscreen blocks both UVA and UVB [56]. However, if sunscreen penetrates the epidermal barrier and gets into contact with living tissue, the DNA damage can be amplified many times, causing damage to living tissue even at very low concentrations (e.g. 10 μmol/L) [57-59].

Skin penetration of organic UV filters such as ethoxylated ethyl-4-aminobenzoate (PEG-25 PABA), benzophenone, benzophenon-3 (oxybenzon), salicylic compounds, octocrylene, octylmethoxycinnamate has been reported [52,60,61]. Skin penetration of metal nanoparticles also causes mistrust of sunscreen products usage [62].

In general, the penetration of rabbit skin > rat > pig > monkey > human, with the pig skin being about 4 times or more and the rat skin up to about 9 times more permeable than human skin for certain compounds [63]. Penetration can also vary depending on the bulk composition of the compound studied. In most safety testing experiments pretreated chemicals isolated from sunscreens are used, but incorporated sunscreen chemicals in cosmetic creams/lotions presented as oil-in-water (O/W) or water-in-oil (W/O) emulsion enhance the penetration of these pretreated chemicals into the skin [60,64]. Also the formulation vehicle in which the sunscreen is presented to the skin has a significant effect on absorption into the skin [65]. Alcohol-based formulations appear to increase sunscreen absorption. In addition, some sunscreen chemicals may enhance the skin absorption of other sunscreens when applied in combination [66].

Some ingredients in sunscreens protect only against direct DNA damage, but increase indirect DNA damage [59,58,67]. It is assumed that this causes an increase in melanoma cases found repeatedly in sunscreen users compared to non-users [68-71]. Other studies have found decreased melanoma risk with increased sunscreen use [72-74]. Discrepancies in reported claims may be caused by differences in the frequency of use, quantities used and the sun protection factor (SPF) of sunscreens. Sunscreens used before likely protected only against UVB, whereas currently sunscreens often have both UVA and UVB protection. Furthermore, although most studies include skin phototype and sun sensitivity, results were not statistically adjusted to account for the sun sensitivity of study participants (i.e. individuals with increased risk for sunburn are more likely to develop melanoma, but they are also most likely to use sunscreens [51].

### Photosensitive drugs

Drug-induced photosensitivity may occur in a variety of ways. Most reactions are generally classified as either phototoxic or photoallergic. Phototoxic reactions are chemically-induced reactions when the drug causes a deeper penetration of UVA light followed by cellular damage. This reaction can be seen with initial exposure to a drug and is perhaps dose-related [75,76]. Photosensitization reactions of drugs lead to the formation of ROS and cause indirect DNA damage [77-80]. It may occur due to topical or systemic drugs (Table 2) [81].

**Table 2:** Some drugs associated with photosensitivity reactions

Frequent	Less frequent
Amiodarone	Antidepressants (tricylic, MAOIs)

NSAIDs	Antifungals

Phenothiazines (particularly chlorpromazine)	Antimalarials

Retinoids	Benzodiazepines

Sulfonamides	Beta-blockers

Tetracyclines (particularly demeclocycline)	Carbamazepine

Thiazides	Griseofulvin

	Oral contraceptives

	Quinine

	Quinolones

	Retinoids

	St John’s Wort

	Sulphonylureas

### Cancer risk of cosmetic ingredients

Cosmetic ingredients are absorbed through the skin. Some chemicals may penetrate the skin in significant amounts, especially when left on the skin for long periods, as in the case of facial makeup. Cancer risks from substances in cosmetic and personal care products have been reported (Table 3) [82].

**Table 3:** Cosmetic ingredients and cancer risk

Cosmetic substance	Risk
DEA (diethanolamine) TEA (triethanolamine)	can result in formation of carcinogenic nitrosamines

Bronopol (2-bromo-2-nitropropane-1,3-diol)	may break down into formaldehyde and also cause the formation of nitrosamines

1,2-Dioxane in surfactants/detergents	contaminated with carcinogenic 1,4-dioxane

Artificial colours (as Blue 1 and Green 3)	Carcinogenic

Hair dyes	dark colours ingredients are carcinogenic

Cosmetic lanolin	can be contaminated with carcinogenic pesticides such as DDT, dieldrin, and lindane, in addition to other neurotoxic pesticides

Talc	Carcinogenic

Silica	may be contaminated with carcinogenic crystalline quartz

Components of temporary tattoos and hair dye ingredients, namely para-aminophenol (PAP) and para-phenylenediamine (PPD), have been reported to be carcinogenic and transformed in human skin [83,84]. However, other studies suggest that consumer or professional exposure to hair dyes poses no carcinogenic or other human health risks [85]. Absorption spectra of these compounds that absorb also UVA /UVB light [86], and possible penetration of these ingredients from dyes, can cause UV-induced indirect DNA damage. According to members of Cosmetic Ingredient Review (CIR), many cosmetic ingredients are used without sufficient data to support safety, especially impurities, UV adsorption, photosensitization, and genotoxicity. Information about cosmetic ingredients such as benzoxiquine, melamine/formaldehyde resin, oxyquinoline, oxyquinoline sulfate etc. should be verified (CIR, 1997, 2006) [87].

### Indoor environment

**Figure 2:**
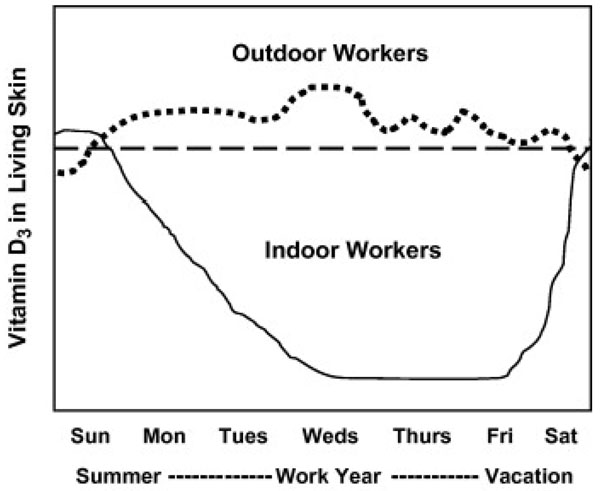
**Cutaneous vitamin D3** The cutaneous vitamin D3 “roller coaster” that indoor workers experience during the workweek and workyear compared to outdoor workers. The curve for indoor workers (solid) rises on some weekends and during most vacations, while for outdoor workers (dotted) cutaneous vitamin D3 remains fairly constant and above the theoretical line for ‘sufficient’ cutaneous vitamin D3. (Godar D.E., 2009).

Outdoor workers can get three to nine times as much solar UVR exposure as indoor workers [88,89]. Paradoxically, outdoor workers have a lower incidence of cutaneous malignant melanoma compared to indoor workers [90-92]. It is supposed that indoor solar UVA exposure, which causes mutations, depletes vitamin D3 in the skin [93]. In the early 20th century, due to life style changes, people tended to stay indoors during the day, which drastically decreased their daily amount of cutaneous vitamin D3. The UV barrier created by window glass separated UVB from UVA, so that the vitamin D making UVB was excluded from our indoor working environment. It is hypothesized that this unnatural UV environment, which existed for decades in buildings and cars, caused cutaneous malignant melanoma incidence to increase steadily about 20–30 years later in the mid-1930s. Increased UVA exposure and decreased cutaneous Vitamin D3 levels may be responsible for the increasing incidence of melanoma [94] (Fig. 2). Melanoma cells can convert vitamin D3 to the hormone, 1,25-dihydroxyvitamin D3, or calcitriol, which causes growth inhibition and apoptotic cell death in vitro and in vivo [94].

### Artificial UV lamps and ionizing radiation

Lamps used for sun tanning emit wavelengths in the short end of the UVA range. Despite claims from the tanning industry, artificial tanning is not a safe or useful way to increase systemic vitamin D levels [93,95]. Many studies indicate a significantly increased risk of cutaneous melanoma subsequent to sunburn/sunlamp exposure, especially among individuals who are young, Caucasian, and female [96-98]. A European study showed that 40 hours of sun bed use resulted in a 55% increased risk for melanoma [99].

More than 40 kinds of skin diseases such as sclerotic skin disease, vitiligo, atopic dermatitis, localized scleroderma etc. can be treated with artificial UVR by three types of phototherapy, namely, broadband UVB phototherapy, narrowband UVB phototherapy, and UVA phototherapy [100-103]. Phototherapy combined with chemicals such as oral methoxsalen (psoralen) in combination with UVA radiation (PUVA) provides a highly effective therapy for psoriasis and many other skin conditions such as vitiligo [104,105,102,101]. However, PUVA is carcinogenic and increases melanoma risk. This risk is greater in patients exposed to high doses of PUVA. It appears to be increasing with the passage of time, and should be considered in determining the risks and benefits of this therapy [106].

It is also suggested that people exposed to ionizing radiation, e.g., nuclear industry workers, subjects near nuclear test blasts, survivors of the atomic bombings of Japan, airline pilots and cabin attendants, recipients of medical radiation, and radiological technicians may be at increased risk of developing melanoma [107].

### Sex hormones and stimulation of melanocytes

Normal and malignant pigment cells are known targets for many hormones. Besides alpha-melanocyte-stimulating hormones and the steroidal hormones estrogen, testosterone, and glucocorticoids, other factors produced by epidermal cells can stimulate melanocytes. Among these factors are the prostaglandins, vitamin D3, ETAF (epidermal cell-derived thymocyte activating factor), and interleukin-1 [108]. A relationship between the biological behaviour of melanoma and sex hormones action has been identified in several areas of research. These observations include different survival prognoses for females and males, the rarity of melanoma incidence in prepubescent children, pregnancy and effects of exogenous hormones [109-111]. Estrogen, estradiol and progesteron receptors have been observed in human melanomas [112-114] and, consequently, melanoma seems to be associated with female hormones.

### Sex

The incidence of malignant melanoma is higher among females than males aged 15-30 years; after age 30, incidence is higher among males [115]. Generally older age and sex are associated with prognostically unfavourable primary cutaneous melanoma. Females have better prognosis than males but this difference disappears after age of 65. Younger patients have more favourable prognosis than older patients; this difference is more pronounced in women [116].

### Pregnancy and melanoma

Melanoma is a major health care problem due to its growing incidence especially in the younger population. In some countries 30% to 35% of female melanoma patients are in their reproductive age [117] and, thus, melanoma can occur in pregnancy ([118,119]. However, recent findings from more recent controlled studies suggest that the data do not support a more advanced stage, thicker tumors, increased metastases to lymph nodes, or a worsened survival of pregnant women diagnosed with localized cutaneous melanoma [120-124]. Pregnancy generally does not increase the subsequent risk of having melanoma and there is no increased risk of melanoma developing during pregnancy [125]. Patients expressing estradiol receptors in melanoma cells have been reported to have a better prognosis [114]. There is little evidence that significant changes in nevi occur during pregnancy [126-128].

### Exogenous hormones

Recent multiple studies showed conflicting results on the association between oral contraceptive use and the development of cutaneous melanoma. While some studies suggest a cumulative dose-dependent increased risk of melanoma with use of estrogens [111], other reports demonstrate that hormone replacement therapy does not appear to enhance the risk for developing melanoma [120]. It is proposed, that estradiol therapy leads to a decrease of proliferation of melanoma cells and an increase in melanogenesis [114]. Endogenous estrogenic metabolite 2-methoxyestradiol was found to have potential preventive/therapeutic use for melanoma growth [129].

### Skin type and melanocytotic hyperpigmentation

Malignant melanoma mainly afflicts people with white skin (the Caucasian population). Odd Ratio for influence by total nevi is 5.37, 95%CI 4.44 to 6.36. Particularly, dysplastic nevi confer much higher risks than most pigment characteristics [130].

Generally, melanoma and its precursor lesions nevi are melanocytotic hyperpigmentation caused by proliferation of active melanocytes. In multiple studies no clear evidence was found to associate melanocytes proliferation with steroid hormones regulation. On the other hand, increased melanin production by existing melanocytes (melanotic hyperpigmentation) is considered to be stimulated also by hormone regulation via melanocytic hormone receptors. Thus, the occurrence of hormone receptors in melanoma cells can be expected because melanoma cells can express pigment.

Melasma is an acquired hypermelanosis, occurring symmetrically on sun-exposed areas of the body. The pigmentation is due to overproduction of melanin by the pigment cells, melanocytes. Lesions are irregular light to dark brown macules and patches, usually involving the forehead, temples, upper lip, and cheeks. Asian and Hispanic females are most commonly affected [131-133]. Other authors found possible role of androgenic hormones in melasma [134]. Typically, melasma happens by increased melanin production by existing melanocytes (melanotic hyperpigmentation) [135]. No increase in the number of melanocytes in melasma areas was noted, but these cells are larger, more dendritic and show increased melanogenesis producing especially eumelanin [136].

During pregnancy, melanocytic activity increases causing hyperpigmentation as observed in the linea nigra (dark line running up the tummy) and the areola and nipple [137-139]. Occurrence of melasma (also known as chloasma) is also found to be associated with estrogen hormones [140,141]: increased expression of estrogen receptors in melasma-affected skin has been demonstrated [141]. Melasma has been reported in 50-70% of pregnant women [142-144] and in non pregnant women who are taking birth control pills [145]. It has been reported to exist only in 10% of men [146]. Sun exposure together with other exogenous factors (such as use of cosmetics and perfumes) are another risk factor for melasma [147]. It has been also thought to arise from endocrine disorders, genetic factors, other medications, nutritional deficiency and hepatic dysfunction [148].

### Family history (germline mutations)

Approximately 5-10% of melanoma occurs in families with hereditary melanoma predisposition. About 40 % of familial melanoma is associated with chromosome 9p [149,150]. Worldwide, approximately 20-40% of kins with familial melanoma harbour germline mutations in the CDKN2A gene, located on chromosome 9p21, which encodes two different proteins, p16INK4 and p14ARF, both involved in regulation of cell cycle progression and induction of senescence. There are geographical variations in the incidence of CDKN2A mutations. The risk of melanoma in CDKN2A mutation carriers varies between populations and is higher in regions with high sun exposure and high incidence of melanoma in the general population [151]. Another melanoma susceptibility gene, CDK4, accounts for only small number of families with germ mutations on chromosome 12q14, encoding a cyclin dependent kinase which normally interacts with p16INK4A [151]. Relative risk to cutaneous melanoma depends on anatomic differences such as body site for skin, or hair colour. These differences could be attributed to gene variability [152].

### DNA polymorphisms/ somatic mutations

A panel of polymorphisms that appears to confer low-to-moderate risk for melanoma has been assessed through functional and genome-wide association studies. Suggested associations between genetic polymorphisms and melanoma were extensively reviewed [153-157]. Vitamin D receptor (VDR) gene SNPs the FokI T allele was associated with increased melanoma risk (OR 1.42, 95% confidence interval CI 1.06-1.91). In a meta-analysis the FokI T allele was associated with increased melanoma risk (OR 1.19, 95%, CI 1.05-1.35), and the BsmI A allele was associated with reduced risk (OR 0.81, 95%, CI 0.72-0.92). However, other study showed opposite results: a significant association between the BsmI VDR polymorphism and increased melanoma risk (OR, 1.30, 95% CI, 1.11-1.53, the population attributable risk 9.2%.). FokI polymorphism did not appear to be associated with such risk (OR, 1.09; 95% CI, 0.99-1.21) [157,158].

Mutations in the cell cycle gene CDKN2A (gene or mono-allelic loss at the locus) were connected with high risk of melanoma but results are not clear [159,160]. The oncogenic mutations in the B-RAF and N-RAS genes constitute the initiating somatic events followed by loss of a major check point gene mainly CDKN2A or in some cases p53 or PTEN [159-161].

The DNA repair process is important in protecting humans from cancer. Multiple DNA repair pathways are able to repair all kinds of DNA damage induced by exogenous and endogenous genotoxic agents, usually in an error-free manner. Recent reviews show links between the DNA repair pathways and cancer, particularly the association between nucleotide excision repair and melanoma development (154, 155, 156). Nucleotide excision repair gene xeroderma pigmentosum variant (XPV) [154], the c.1783G, p.595V alleles were associated with melanoma (OR 1.86 CI 1.27-2.71, and OR 1.84 1.29-2.63 respectively). XPD/ERCC2 SNP rs1318, variant C allele was associated with slightly increased melanoma risk (OR = 1.12, 95% CI 1.03-1.21, population attributable risk = 9.6%) [156].

Some of the genetic variants in the DNA repair gene XRCC1 have also been associated with melanoma. Patients with variant genotype had better overall survival. MC1R variants were associated with susceptibility to basal cell carcinoma of skin and there is an interaction with host factors and the XRCC3 gene [162]. ASIP and TYR pigmentation variants are also associated with cutaneous melanoma and basal cell carcinoma. These results suggest that both nucleotide as well as base excision repair deficiency may contribute to the development of cutaneous melanoma. However, recent results show that the repair of major DNA damage caused by cancer drugs is efficient in metastatic melanoma [156]. This implies that in addition to genome association studies, more research on DNA repair and cell cycle regulation is needed.

## Conclusions

Cutaneous melanoma is a complex, heterogeneous cancer that is increasing in incidence. Multiple studies have identified major host and environmental risk factors for melanoma. The predominant environmental risk factor is exposure to UV radiation. Geographical and individual factors such as sex, skin type (particularly dysplastic nevi) and life style – outdoor/indoor life, sunburn, vitamin D and antioxidant protections are considered to be risk factors. Additionally, exposure to other environmental factors such as sunscreen, photosensitising drugs, and exogenous hormones may also increase the risk of melanoma. Recently, the association between rare high risk susceptibility genes and common polymorphic genes and development of melanoma risk have been identified. However, more epidemiological as well as mechanistic studies are needed to understand the causal mechanisms of melanoma development.

## Competing interests

None declared

## Authors' contributions

KV, DB, and MD conceived and designed the review, collected the data and drafted the manuscript. SL contributed to the conception of the review and its design. AB is a HENVINET project coordinator and contributor to Framework development. All authors read and revised the final version of the manuscript.
